# *NT5C2* methylation regulatory interplay between DNMT1 and insulin receptor in type 2 diabetes

**DOI:** 10.1038/s41598-020-71336-9

**Published:** 2020-09-30

**Authors:** Yng-Tay Chen, Wei-De Lin, Wen-Ling Liao, Ya-Ching Tsai, Jiunn-Wang Liao, Fuu-Jen Tsai

**Affiliations:** 1 Graduate Institute of Food Safety, College of Agriculture and Natural Resources, National Chung Hsing University, Taichung, Taiwan; 2 Human Genetic Center, Department of Medical Research, China Medical University Hospital, China Medical University, Taichung, Taiwan; 3 School of Post Baccalaureate Chinese Medicine, China Medical University, Taichung, Taiwan; 4 Graduate Institute of Integrated Medicine, China Medical University, Taichung, Taiwan; 5 Center for Personalized Medicine, China Medical University Hospital, Taichung, Taiwan; 6 Graduate Institute of Veterinary Pathobiology, National Chung Hsing University, Taichung, Taiwan; 7 School of Chinese Medicine, China Medical University, Taichung, Taiwan; 8 Department of Health and Nutrition Biotechnology, Asia University, Taichung, Taiwan

**Keywords:** Predictive markers, Epigenetics

## Abstract

Epigenetics alternation of non-genetic variation and genome-wide association study proven allelic variants may associate with insulin secretion in type 2 diabetes (T2D) development. We analyzed promoter DNA methylation array to evaluate the associated with increased susceptibility to T2D (30 cases, 10 controls) and found 1,091 gene hypermethylated in promoter regions. We performed the association study of T2D and found 698 single nucleotide polymorphisms in exon and promoter sites by using 2,270 subjects (560 cases, 1,710 controls). A comparison of DNA hypermethylation and gene silencing of mouse T2D results in our T2D patients’ results showed that the 5′-nucleotidase, cytosolic II (*NT5C2*) and fucosyltransferase 8 (*FUT8*) genes were strongly associated with increased susceptibility to T2D. DNA hypermethylation in promoter regions reduced *NT5C2* gene expression, but not *FUT8* in T2D patients. NT5C2 protein expression was decreased in pancreatic β-cells from T2D mice. Transient transfection *NT5C2* into RIN-m5F cells down-regulated DNA methyltransferase I (DNMT1) expression and up-regulation of the insulin receptor. Moreover, *NT5C2* knockdown induced in DNMT1 overexpression and insulin receptor inhibition. Taken together, these results showed that NT5C2 epigenetically regulated insulin receptor in patients and mice with T2D, and maybe provide for T2D therapy strategy.

## Introduction

The increasing prevalence of type 2 diabetes (T2D) is a global health problem. T2D can lead to various complications, including diabetic nephropathy, retinopathy, stroke, neuropathy, atherosclerosis, and hypertension, affecting patient quality of life and mortality. T2D is a disease with multifactor whose onset and development depend on not only genetic factors but also other factors, for example, epigenetics regulations. Epigenetic mechanisms may play an important role in T2D development^1,2^. T2D is characterized by hyperglycemia, pancreatic β-cell dysfunction, decreased insulin signaling action, and increased hepatic glucose formation^3–5^. In the pancreas, DNA methylation is involved in regulating de novo β-cell formation^6^. Previously studies have indicated that epigenetic factors conduce to the onset of T2D^7–12^. However, the mechanisms how DNA methyltransferase I (DNMT1) regulate T2D are unclear, and the roles of DNMT1 in T2D onset and development have not been clarified.

Investigation of the associations of DNA methylation in the peripheral blood may facilitate the identification of biomarkers for noninvasive early disease detection in epidemiological studies^13^. DNA methylation, may be associated with regulation of the risk of many pathologies, such as T2D. DNA methylation can also affect the relationship between T2D and environmental exposure^14^.

Evaluation of epigenetics difference by Genome-wide DNA methylation arrays have become popular to assess disease progression. Currently, T2D onset and development biomarker identify from by epigenetics regulation still unclear in Taiwan Han Chinese. We using a DNA methylation array results to comparison with single nucleotide polymorphisms (SNPs) to looking for biomarker. Moreover, how DNMT1 affects the insulin signaling pathway, we examined epigenetic changes in patients with T2D, a mouse model of T2D, and in vitro transfections experiences. Our results provided the important role of DNA methylation in the T2D disease pathway.

## Results

### DNA promoter methylation in patients with T2D

We performed a promoter DNA methylation array of samples from 30 patients with T2D and 10 healthy controls, and clinical characteristics of the subjects showed in Table 1. DNA methylation array data are accessible via the Gene Expression Omnibus (GEO) database, accession number GSE81868 (https://www.ncbi.nlm.nih.gov/geo/query/acc.cgi?acc=GSE81868). Analysis of DNA methylation status was carried out using model-based analysis of tiling-arrays (MAT) levels, found 1,091 genes were hypermethylation in promoter regions (Table S4).

**Table 1:** Clinical characteristics of the subjects for DNA methylation array.

	T2D	Controls
Number	30	10
Male/female (%)	50/50	50/50
Age at study (years)	61.9 ± 10.4	59.3 ± 8.8
BMI (kg/m^2^)	25.5 ± 4.6	23.5 ± 3.8
HbA1c (%)	8.4 ± 2.0	5.3 ± 0.6
Fasting plasma glucose (mg/dL)	141.4 ± 34.7	–
Diabetic duration (years)	13.1 ± 3.9	–
SBP (mmHg)	131.6 ± 18.4	128.8 ± 18.8
DBP (mmHg)	76.7 ± 11.6	75.5 ± 11.0

### SNPs in T2D

We performed DNA promoter and exonic sites of susceptibility SNPs to T2D from subjects of 560 T2D cases and 1,710 controls, clinical characteristics of the subjects shown in Table 2. We found 698 SNPs at promoter and exon regions associated with T2D (Table S1). There are 33 genes not only with SNPs susceptibility to T2D but also DNA promoter hypermethylation (Table 3).

**Table 2:** Demographic and clinical characteristic for subjects who were used for identified 698 SNPs.

	T2D (N = 560)	Control (N = 1,710)	P*
Male (%)	52.1	50	0.379
Age at study	63.2 ± 11.5	47.4 ± 10.7	< 0.001
Age at diagnosis	50.5 ± 13.1	–	
BMI (kg/m^2^)	25.7 ± 4.2	24.3 ± 3.6	< 0.001
HbA1c (%)	7.9 ± 1.8	5.8 ± 0.9	< 0.001
Fasting plasma glucose (mg/dL)	145.6 ± 66.5	96.3 ± 21.3	< 0.001
SBP (mmHg)	141.4 ± 19.7	113.8 ± 16.4	< 0.001
DBP (mmHg)	79.5 ± 13.3	71.8 ± 11.2	< 0.001

Values are presented as N (%) or mean ± SD.

*T2D* type 2 diabetes, *BMI* body mass index, *HbA1c* hemoglobin A1c, *SBP* systolic blood pressure, *DBP* diastolic blood pressure.

*P value for chi square test or two-sample independent *t* test.

**Table 3:** DNA methylation and SNP matching gene list in patients with T2D.

Transcript ID	Gene symbol	MAT-score	Description
NM_199427	*ZFP64*	9.10027	ZFP64 zinc finger protein
NM_000814	*GABRB3*	8.33046	Gamma-aminobutyric acid type A receptor beta3 subunit
NM_015335	*MED13L*	7.13939	Mediator complex subunit 13 like
NM_001282773	*RGS7*	6.8489	Regulator of G-protein signaling 7
NM_006699	*MAN1A2*	5.49222	Mannosidase alpha class 1A member 2
NM_001289905	*IL17RA*	5.48686	Interleukin 17 receptor A
NM_001401	*LPAR1*	5.20981	Lysophosphatidic acid receptor 1
NM_001035235	*SRA1*	5.17213	Steroid receptor RNA activator 1
NM_001134373	*NT5C2*	5.13721	5′-Nucleotidase, cytosolic II
NM_001282225	*CECR1*	5.11905	Cat eye syndrome chromosome region candidate 1
NM_001258282	*LINGO2*	4.95801	Leucine rich repeat and Ig domain containing 2
NM_203349	*SHC4*	4.78135	Src homology 2 domain containing family member 4
NM_001286401	*TMEM217*	4.60762	Transmembrane protein 217
NM_001243042	*HLA-C*	4.46832	Major histocompatibility complex, class I, C
NM_001102654	*NTF3*	4.45068	Neurotrophin 3
NR_033984	*LOC400548*	4.35767	Uncharacterized LOC400548
NM_001166412	*SMOC2*	4.33842	SPARC related modular calcium binding 2
NM_018429.2	*BDP1*	4.28144	B double prime 1, subunit of RNA polymerase III transcription initiation factor IIIB
NM_004480	*FUT8*	4.19785	Fucosyltransferase 8
NM_182511	*CBLN2*	4.18523	Cerebellin 2 precursor
NM_001243108	*PLD2*	4.17228	Phospholipase D2
NM_002318	*LOXL2*	4.13622	Lysyl oxidase like 2
NM_005215	*DCC*	4.11725	DCC netrin 1 receptor
NM_001010848	*NRG3*	4.11385	Neuregulin 3
NM_001195001	*PTPRU*	4.08896	Protein tyrosine phosphatase, receptor type U
NM_016529	*ATP8A2*	4.05786	ATPase phospholipid transporting 8A2
NM_002263	*KIFC1*	4.00384	Kinesin family member C1
NM_001105579	*SYNDIG1L*	3.93368	Synapse differentiation inducing 1 like
NM_001759	*CCND2*	3.78164	Cyclin D2
NM_001166058	*RXFP2*	3.71546	Relaxin/insulin like family peptide receptor 2
NM_022469	*GREM2*	3.69799	Gremlin 2, DAN family BMP antagonist
NM_153810	*CACUL1*	3.55334	CDK2 associated Cullin domain 1
NM_001145159	*INTS9*	3.5282	Integrator complex subunit 9

### DNA promoter hypermethylation and gene expression in T2D mice

We next performed promoter DNA methylation array analysis using KK-Ay mice with T2D and KK control mice. DNA methylation array data are accessible via the GEO database, accession number GSE100677 (https://www.ncbi.nlm.nih.gov/geo/query/acc.cgi?acc=GSE100677). The primary data are accessible via the GEO database, accession number GSE101879 (https://www.ncbi.nlm.nih.gov/geo/query/acc.cgi?acc=GSE101879). We found 260 genes showing DNA promoter hyper-methylation and gene silencing (Table S2).

### Comparison of epigenetic changes in genes in humans and mice with T2D

**Figure 1:**
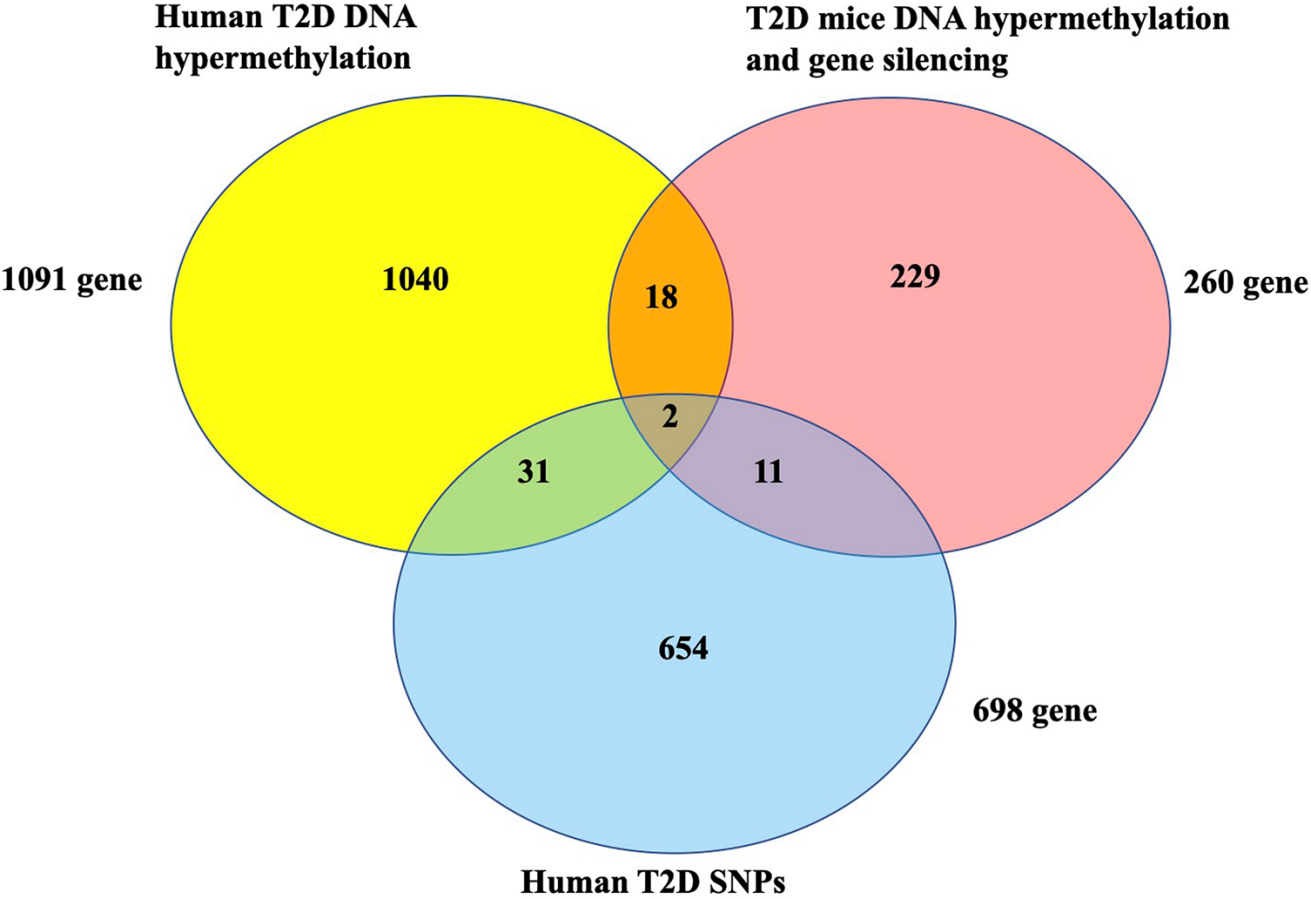
The intersection between human T2D DNA methylation, human T2D SNPs, and T2D mice gene silence following DNA methylation. We found 1,091 genes were hypermethylation in promoter regions from T2D patients. 698 promoter and exon regions SNPs associated with human T2D. 260 genes showing DNA promoter hypermethylation and gene silencing in T2D mice.

We compared the 33 genes from human T2D to 260 genes from mice T2D. We identified the 5′-nucleotidase, cytosolic II (NT5C2) and fucosyltransferase 8 (FUT8) genes which were associated with susceptibility to T2D not only SNPs but also DNA promoter hypermethylation in human T2D, and gene expression inhibition following by DNA methylation in T2D model mice (Fig. 1). The positions of NT5C2 and FUT8 SNPs were located on chr10:103089387 and chr14:65900969, respectively. NT5C2 and FUT8 DNA promoter methylation MAT-score were 5.13721 and 4.19785, respectively.

### NT5C2 and FUT8 mRNA expression from peripheral blood of T2D patients

**Figure 2:**
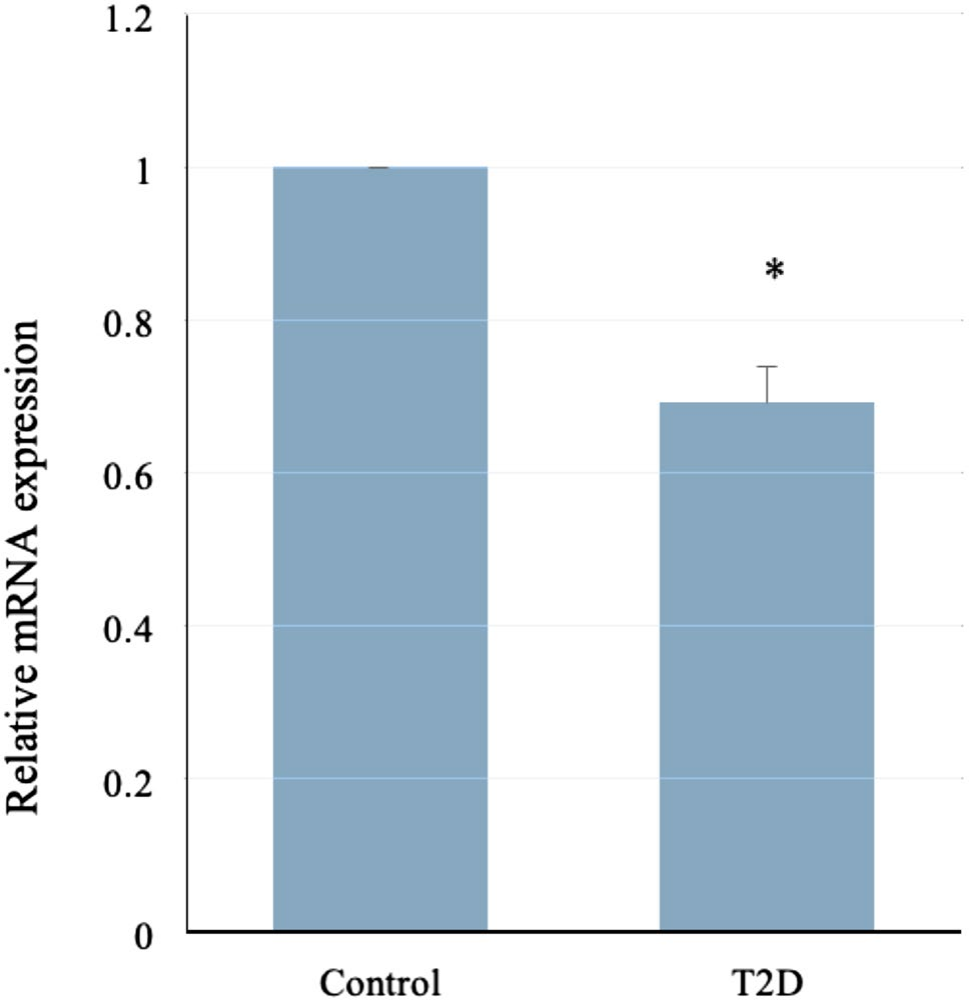
*NT5C2* mRNA was downregulated in patients with T2D. NT5C2 mRNA expression is significantly lower in patients with T2D (N = 94) than in control (N = 98). The relative mRNA levels in patients is 0.67 ± 0.03 versus healthy control (*P* < 0.05, SPSS software 15.0 for windows).

Further analysis of peripheral blood samples from 94 patients with T2D and 98 healthy controls, the clinical characteristics of the subjects showed in Table 4. The subjects HbA1c were 8.0 ± 1.5%, diabetic duration was 8.7 ± 6.5 years. And the group showed DNMT1 gene overexpression in our previously data^18^. Result showed that relative *NT5C2* mRNA levels were lower in patients with T2D than in controls (1 vs 0.67 ± 0.03, respectively; *P* < 0.05; Fig. 2). However, *FUT8* mRNA levels in T2D were showed no significant difference with healthy controls (Figure S2).

**Table 4:** Clinical characteristics of the subjects.

	T2D	Controls
Number	94	98
Male/female (%)	50/50	50/50
Age at study (years)	56.7 ± 12.1	55.8 ± 13.5
BMI (kg/m^2^)	25.1 ± 3.8	23.0 ± 3.3
HbA1c (%)	8.0 ± 1.5	5.0 ± 0.4
Fasting plasma glucose (mg/dL)	148.5 ± 29.7	–
Diabetic duration (years)	8.7 ± 6.5	–
SBP (mmHg)	135.4 ± 16.1	121.5 ± 17.5
DBP (mmHg)	79.6 ± 14.3	74.4 ± 18.6

### Effects of NT5C2 on BMI

In Taiwan, BMI categories included underweight (BMI < 18.5 kg/m^2^), normal weight (18.5 ≤ BMI < 24 kg/m^2^), overweight (24 ≤ BMI < 27 kg/m^2^), obesity (BMI ≥ 27 kg/m^2^). We analyzed those 698 SNPs associated with T2D among subjects with BMI ≧ 24. Results show that 291 SNPs, including rs12573221, were associated with T2D in the overweight subgroup (BMI ≧ 24, n = 1,179) (Table S1).

We further analyzed the SNPs near the NT5C2 gene which is associated with BMI in Asian and European populations (Table S3). The information was extracted from the GWAS catalog. rs11191560 was associated with BMI in European population (OR = 0.0288, 95% CI 0.017–0.041) (www.ncbi.nlm.nih.gov/pubmed/28443625) and rs11191580 was associated with BMI in Asian population (OR = 0.0295, 95% CI 0.019–0.04) (www.ncbi.nlm.nih.gov/pubmed/24861553). The identified SNP, rs12573221, was not highly linkage with those two SNPs (R^2^ < 0.2).

### NT5C2 was inhibited in pancreatic β-cells in mice

**Figure 3:**
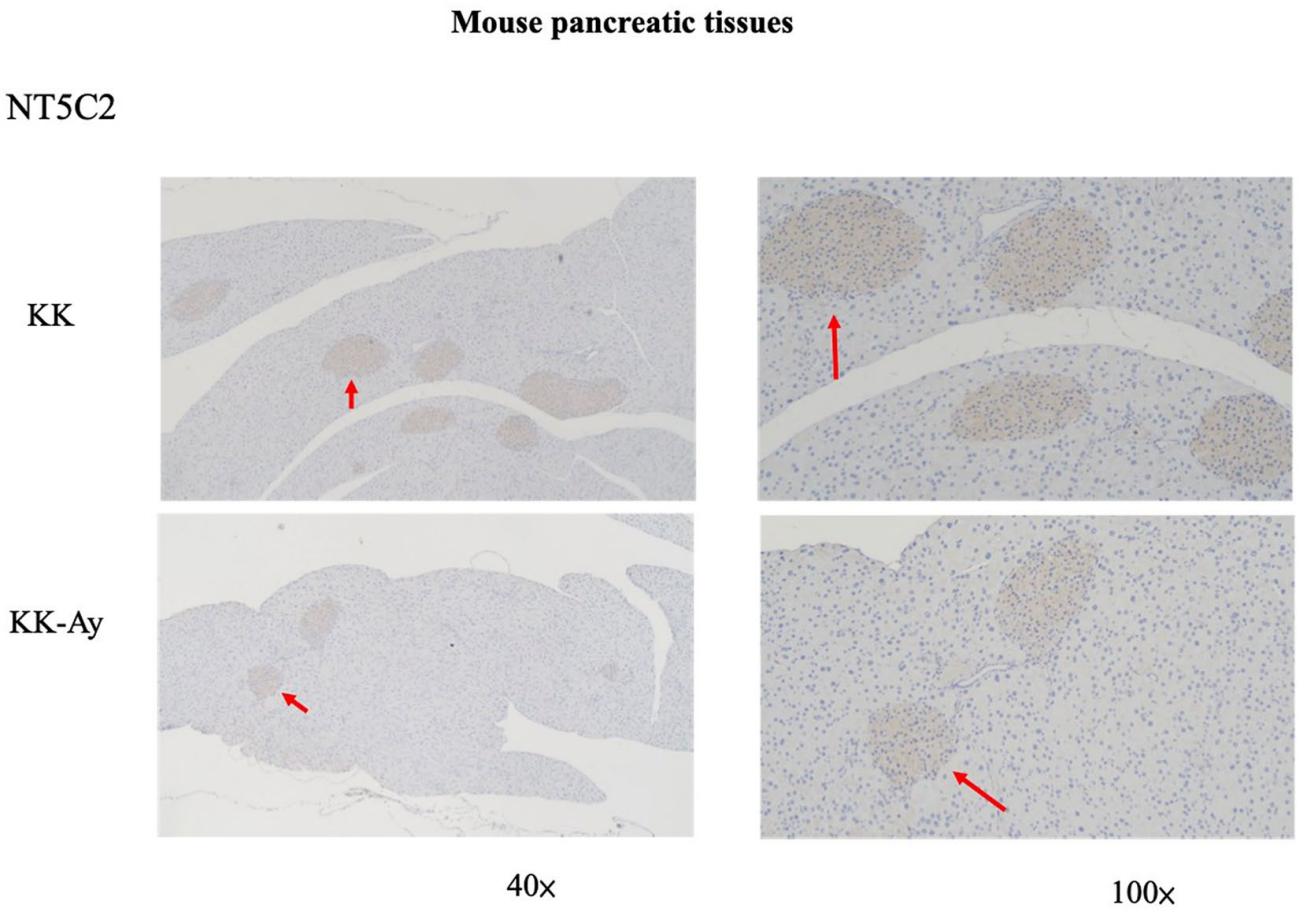
NT5C2 expression was inhibited in the pancreas of T2D model mice. Immunocytochemistry staining of pancreatic tissues show NT5C2 protein expression in KK and KK-Ay mice at 42 weeks of age. The red arrowheads are pointing at the pancreas β-cells and showing NT5C2 protein expression. The brown color depicts NT5C2-positive cells in the pancreas. Representative images of anti-NT5C2 IHC on the pancreas, the NT5C2 expression was higher in KK mice than KK-Ay mice. The red arrowheads are pointing at NT5C2-positive cells.

We used a mouse model of T2D to further elucidate the role of NT5C2 in T2D. In KK-Ay mice (42 weeks old) with late-stage T2D, insulin resistance was found to be associated with hypertrophy in pancreas islets and degranulation of β cells. Moreover, NT5C2 protein expression was inhibited in pancreatic, especially in β-cells, in T2D mice versus control mice (Fig. 3).

### NT5C2 affected the insulin signaling pathway via DNMT1

**Figure 4:**
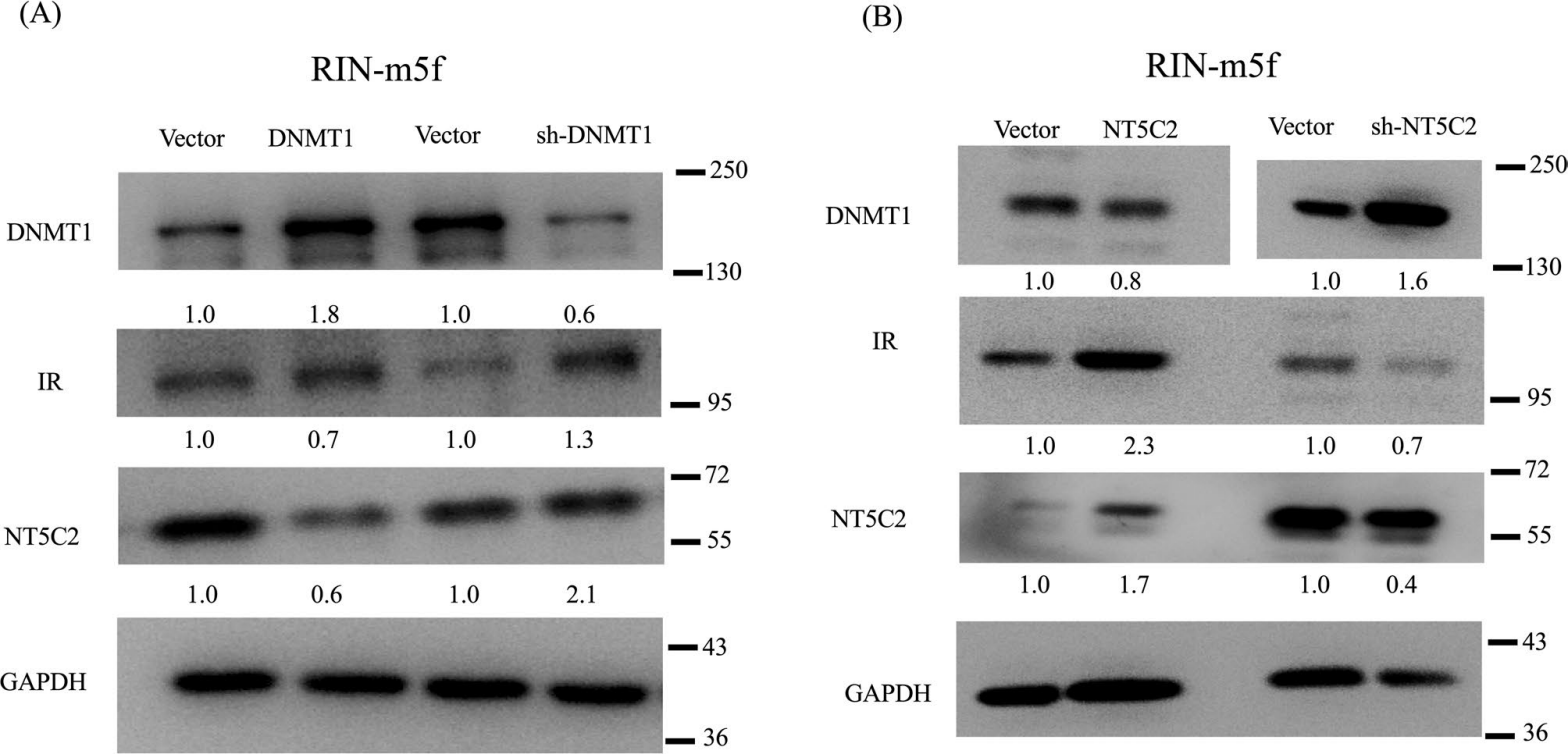
The *NT5C2* gene was epigenetically regulated in the insulin-signaling pathway. (**A**) RIN-m5F cells were transfected with pvDNA-DNMT1 or shRNA DNMT1 for 48 h, and the effects of DNMT1 overexpression on NT5C2 and insulin receptor (IR) inhibition or the effects of DNMT1 knockdown on NT5C2 overexpression and IR expression were analyzed. (**B**) RIN-m5F cells were transfected with pvDNA3-NT5C2 or shRNA NT5C2 for 48 h, and the effects of NT5C2 overexpression on DNMT1 inhibition and induction of IR expression or the effects of NT5C2 knockdown on DNMT1 overexpression and IR inhibition were analyzed. The numbers in the figure indicated the Image J results of protein expression versus vector control. The samples drive from the same experiment and the blots were processed in parallel. We were cutting the PVDF membrane according to molecular weight and reacted with DNMT1, NT5C2, IR, and GAPDH antibodies separately. The staining of GAPDH was used as a loading control. The original and fuller-length image of blots is presented in Supplementary Figure S1.

**Figure 5:**
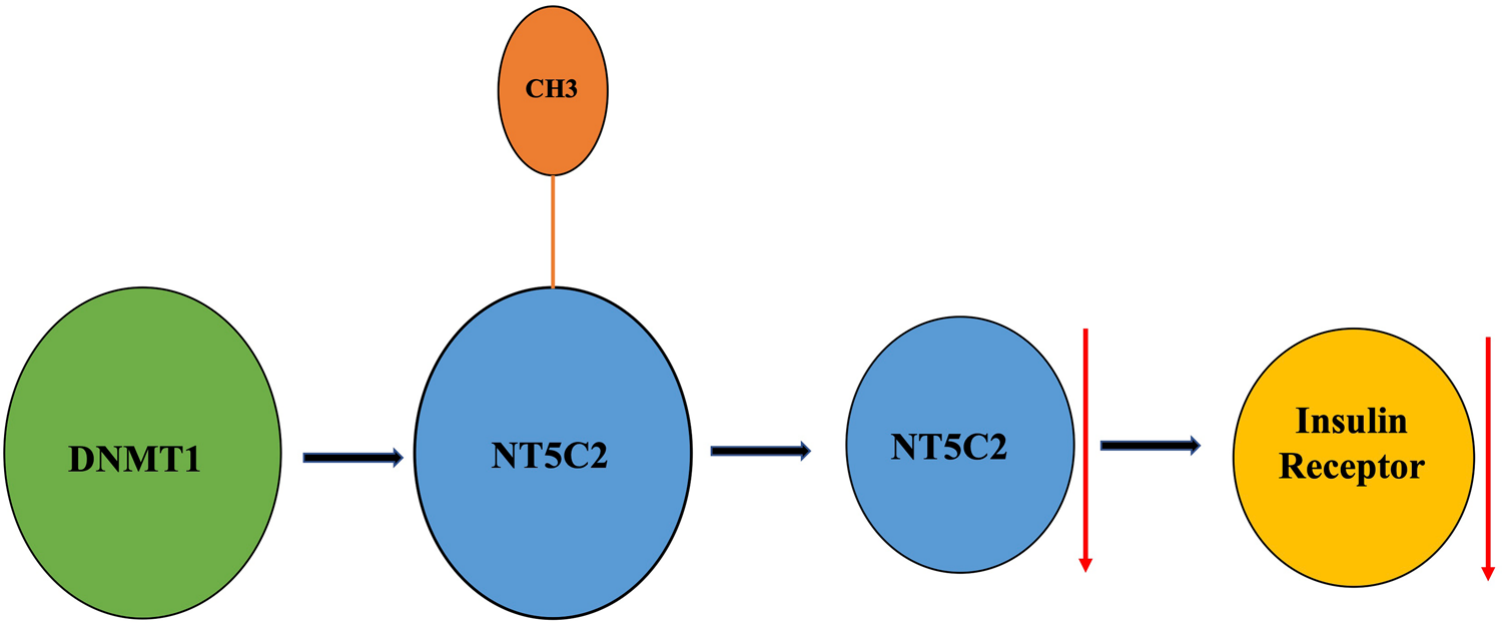
DNMT1 epigenetically regulates NT5C2. DNMT1 overexpression induced NT5C2 DNA hypermethylation and leading to NT5C2 gene silencing and insulin receptor inhibition. Overexpression of NT5C2 caused DNMT1 expression inhibition and insulin receptor activation.

The role of DNMT1 in modulating the effects of NT5C2 in the insulin signaling pathway is unclear. Therefore, we used an in vitro transient transfection model to analyze the effects of NT5C2 expression on insulin signaling. A plasmid containing human DNMT1 or NT5C2 was constructed and transiently transfected into RIN-m5F cells. The results indicated that DNMT1 overexpression caused NT5C2 and insulin receptor inhibition (Fig. 4A), whereas knockdown of DNMT1 expression by short hairpin RNA (shRNA) in RIN-m5F cells induced NT5C2 and insulin receptor overexpression. In contrast, NT5C2 overexpression caused DNMT1 inhibition and insulin receptor overexpression, whereas NT5C2 knockdown induced DNMT1 overexpression and insulin receptor inhibition (Fig. 4B). These results suggesting that NT5C2 was involved in the insulin-signaling pathway and was affected by DNMT1 (Fig. 5).

## Discussion

There are one in three T2D patients with the microvascular complications at the time of diagnosis. The complications of T2D included cardiovascular disease, foot disease, retinopathy, and kidney disease^15^. Diabetic nephropathy and diabetic retinopathy include two major microvascular complications and occur with a high concordance rate in T2D. Previously study has suggested that these two complications might go through the same pathogeneses, such as glucose metabolism, angiogenesis, inflammation, and oxidative stress. The medications for T2D usually used oral hypoglycemic drugs and insulin injections. These complications may development for 5 years before the diagnosis of T2D^16^.

GWAS and DNA methylation array data can show markers that may explain onset and development of T2D population. Analysis of DNA methylation may also have uses as a diagnostic and therapeutic tool in patients with T2D^17–21^. In this study, we found that the *NT5C2* and *FUT8* genes were hypermethylated in both patients and mice with T2D, but only *NT5C2* showed downregulation of mRNA expression in T2D patients. DNA methylation arrays have made better the study of DNA methylation, but little CpG methylation site are limited^19^. The role of epigenetics regulation in T2D pathogenesis needed to analysis, epigenetic regulations may predict to T2D onset and development^18,21^. Human, animal model, and in vitro T2D model are also needed, these models may explanation how epigenetic regulations affect T2D onset and development^20^.

We reported that the DNMT1 was associated with susceptibility to T2D patients^18,21^. DNMT1 was significantly expression higher in T2D than in controls^18^. DNMT1 with C allele single nucleotide polymorphism (rs78789647), promoter hypermethylated that affects insulin signaling pathway^21^. In this study, *NT5C2* was regulated by DNMT1 and affected the insulin receptor. NT5C2 is an enzyme that dephosphorylate noncyclic nucleoside monophosphates to nucleoside and inorganic phosphate^22^. This enzyme is critical for control of energy balance, metabolic regulation, and cell replication^23^. NT5C2 is involved in maintaining cellular nucleotide and nucleoside levels by catalyzing the hydrolysis of AMP to adenosine, and hydrolysis IMP to inosine^24^. NT5C2 is expressed in skeletal muscle from tissue expression study^23^. *NT5C2* in human myotubes, increases AMP-activated protein kinase (AMPK), acetyl-CoA carboxylase phosphorylation, and promotes lipid oxidation and glucose transport. The NT5C2 and AMPK activity in T2D and obesity may play an important role in the regulation of insulin action and lipid metabolism in skeletal muscle^23^.

NT5C2 is present in the brain, heart, skeletal muscle, erythrocytes, spleen, testis, fibroblasts, and endothelial cells^23^. We checked the NT5C2 protein expression of the brain, heart, muscle, spleen, and testis by immunohistochemistry staining. Since the NT5C2 were low activity presentations in these tissues, there was no significant difference between KK and KK-Ay mice (data not showed). KK-Ay mice with the lethal yellow obese (A^y^) mutation and develop diabetes of polygenic origin, showing severe obesity, hypertriglyceridemia, hyperglycemia, hyper-insulinemia, and insulin function loss by 42 weeks of age^18,21,25,26^. In our results, pancreatic β-cell mass was proliferation, and NT5C2 protein expression was inhibited in T2D mice’s pancreatic β-cell. Our previously results showed DNMT1 was significantly overexpression in T2D mice’s pancreatic β-cells^20,21^. Previously study have showed the importance of DNA methylation in pancreatic islet function^10^. In these studies, researchers identified the promoters of the insulin gene (*INS*)^27^; pancreatic and duodenal homeobox 1 (PDX1)^28^, which encodes a transcription factor important for both pancreatic development^29^ and the function of mature β-cells^30^; PPARG coactivator 1 alpha (*PPARGC1A*)^31^; and glucagon-like peptide 1 receptor (GLP1R)^32^, which stimulates insulin secretion and protects β-cell proliferation, as hypermethylated in islets from donors with T2D compared with those in islets from nondiabetic donors^33^. A hypermethylation was associated with reduced mRNA expression of the respective gene in pancreatic islets and with higher glycated HbA1c, suggesting the role in β-cell disturbs in T2D. High levels of glucose were also found to directly increase DNA methylation of *Pdx1* and *Ins* in clonal β-cells^27,28^.

## Conclusions

In conclusion, the present study demonstrated the existence of a specific methylome map associated with genome-wide association studies in T2D. To the best of our knowledge, this study is the first to investigate the DNA methylation results and candidate genes from a combination of GWAS to determine NT5C2 gene is associated with T2D development.

## Methods and materials

### DNA methylation microarray analyses for human T2D and control

Genomic DNA extraction according to the manufacturer’s method (Qiagen, Valencia, CA, USA) and our previously methods^18^, peripheral blood leukocytes were obtained from T2D cases and healthy control. DNA (50 ng) amplified by using a Whole Genome Amplification Kit (cat. no. WGA4; Sigma, St. Louis, MO, USA), and 7.5 μg of reaction DNA was fragmented and labeled. Reaction was using the Affymetrix GeneChip Promoter 1.0R array. Arrays were stained with streptavidin–phycoerythrin, and scanned on an Affymetrix GeneChip Scanner 3000. The 40 samples (30 T2D cases were sample randomly from the 94 T2D patients and 10 healthy controls were sample randomly from the 98 healthy controls) which pass QC according principal component analysis. The methylation regions of the T2D and the controls were compared using the Model-based Analysis of Tiling-array (MAT) calculation (*P* < 0.001)^34^.

### Subject participants

A total of 2,270 subjects, including 560 T2D cases and 1,710 controls were used for GWAS in this study. T2D subjects were enrolled from China Medical University Hospital (CMUH)^35^ and were diagnosed according to the American Diabetic Association Criteria by using medical records and fasting plasma glucose levels. Ocular history data collected were from the questionnaires. For each patient, SBP, DBP and BMI were determined, and blood samples were collected by venipuncture for genomic DNA isolation and serological tests, including fasting glucose and HbA1c, at the time of participate the study at clinical office of CMUH. The healthy controls were selected from the Taiwan BioBank. All of the participating T2D cases and controls were of Han Chinese origin in Taiwan. Genomic DNA extracted from peripheral blood leukocytes with the Genomic DNA kit (Qiagen, CA, USA.) was genotyped by using Affymetrix Axiom genome-wide TWB array according to standard quality control procedures. The information of SNPs on promoter region and exonic sites on the chip were extracted.

This study was approved by the Human Studies Committee of China Medical University Hospital (CMUH103-REC2-071) and informed consent was obtained from all participants. The study was conducted in accordance with the tenets of the Declaration of Helsinki.

### Quantitative real-time polymerase chain reaction (qRT-PCR) analysis for T2D Patients

There were 192 subjects were recruited from China Medical University Hospital, Taichung, Taiwan. The 94 subjects were diagnosed as T2D according to medical records and fasting plasma glucose levels using American Diabetic Association Criteria. The criteria for 98 healthy controls were no diagnostic history of T2D. The HbA1C values were lower than 6%, and BMI less than 32 kg/m^2^. The healthy controls were comparable with respect to BMI, gender, age at study, and level of HbA1c. Total RNA was isolated from human blood using a High Pure RNA Isolation Kit (Roche, Mannheim, Germany) according to the manufacturer’s instructions. cDNA was synthesized from 1 µg total RNA using a High Capacity cDNA Reverse Transcription Kit (Applied Biosystems, Foster City, CA, USA) in a 20-µL reaction volume, according to the manufacturer’s instructions. cDNA was diluted to 10 ng/L, and 1 µL cDNA was used for each qRT-PCR assay in a final reaction volume of 10 µL. For quantification of gene expression with the ABI ViiA 7 Real-Time PCR System (Applied Biosystems), FastStart Universal SYBR Green Master mix (Roche) was used^16^. Primer sequences were as follows: *NT5C2* sense, 5′-TGCAGCATCTTTCATCAACC-3′ and antisense, 5′-TGCTCCAC CGTTGATTCAT-3′; *GAPDH* sense, 5′-CAGCCTCAAGATCA TCAGCA-3′ and antisense, 5′-TGTGGTCATGAGTCCTTCCA-3′.

### Animals

KK and KK-Ay (KK.Cg-*A*^*y*^/J) mice were obtained from Jackson Laboratories (Bar Harbor, ME, USA). Forty-two-week-old mice were used for analyses. Five mice were housed in one cage and fed lab chow ad libitum (LabDiet 5k52, St. Louis, MO, USA). The animal room was with controlled temperature from 22 to 25 °C, the humidity was 50 to 70%, and 12-h light and 12-h dark. The experiment according to the criteria for the care and use of KK and KK-Ay mice laid out in the ‘‘Guidebook for the Care and Use of Laboratory Animals’’^36^. This study was approved by the Institutional Animal Care and Use Committee (IACUC) of China Medical University (IACUC: 102-217).

### T2D mice Gene expression microarray analysis

Total RNA was isolated from mice liver tissue using the RNA isolation kit according to the manufacturer’s instructions (High Pure, Roche, Mannheim, Germany) as our previously methods^18^. And briefly describe below, cDNA was synthesized from RT kit (Ambion WT, Life Technologies) according to the manufacturer’s instructions. The mix was hybridized to a GeneChip Mouse Exon 1.0 ST Array (Affymetrix) in an oven overnight. Probe intensities were detected by using an Affymetrix GeneChip Scanner 3000 7G, and probe cell intensity data (CEL) were analyzed by using Affymetrix Expression Console software version 4.0 to generate CHP files with the Robust Multichip Analysis (RMA)-sketch algorithm processes. The transcript structure levels for gene and exon level analyses, which restricts analysis to exon-level probe sets that map to BLAT sequences of mRNAs with noted coding sequence regions. Genes and exons expression difference were identified by Transcriptome Analysis Console 2.0.0.9 software (Affymetrix).

### Immunohistochemistry (IHC)

For paraffin tissue slice deparaffinization and rehydration, the slices were incubated in 3% H_2_O_2_ solution for 30 min. Then the slices were boiling in 0.01 M citrate buffer for 20 min for antigen retrieval, and washed in 50 mM Tris-HCl (pH 7.6) with 0.05% Tween for 2 min. Slices were incubated with 5% nonfat dry milk for 30 min for block nonspecific binding. The slides were then hybrid with anti-NT5C2 antibodies (1:200, GTX105719, GeneTex) for 1 h, then incubated with secondary antibody, and incubated with a peroxidase-labeled streptavidin–biotin complex and diaminobenzidine substrate to demonstrate the NT5C2 labeled cells.

### Transfection

RIN-m5F rat pancreatic β-cells were purchased from Food Industry Research and Development Institute (Hsinchu, Taiwan). Cells were seeded at 150,000 cells/well in six-well culture plates and incubated until the culture reached 50–80% confluence. Cells were then transfected with the empty pCMV3-untagged vector, pCMV3-DNMT1 construct, or pCMV3-NT5C2 construct. Lentiviral expression system for NT5C2 or DNMT1 shRNA (provided by the National RNAi Core Facility, Academia Sinica, Taiwan) using Xfect Transfection Reagent (Clontech, Palo Alto, CA, USA), according to the manufacturer’s instructions and methods^16^. Total protein was isolated from the cells after 48 h reaction.

### Protein extraction and western blotting

RIN-m5F cells were homogenized in ice-cold RIPA lysis buffer (Millipore, Temecula, CA, USA), protease inhibitor, and phosphatase inhibitor (FIVEphoton, San Diego, CA, USA). The homogenate was incubated on ice for 1 h at 4 °C and centrifuged at 12,000×*g* for 30 min at − 20 °C. The supernatant was used for western blotting. Proteins sample of the supernatant were used by SDS PAGE using 10% acrylamide gels and transferred to PVDF membranes. PVDF membranes were cut according to molecular weight and reacted with antibodies separately. The samples drive from the same experiment and the blots were processed in parallel. Membranes were incubated in 5% skill milk with 0.1% Tween-20 in Tris-buffered saline, followed by incubation with rabbit anti-NT5C2 (1:1,000; GeneTex, Irvine, CA, USA), anti-DNMT1 (1:1,000; LifeSpan BioSciences), anti-insulin receptor (1:1,000; GeneTex), or anti-GAPDH (1:5,000; GeneTex) polyclonal primary antibodies. Membranes were then incubated with HRP-conjugated goat anti-rabbit IgG (1:5,000; Jackson ImmunoResearch, West Grove, PA, USA) secondary antibodies. Proteins were observed using SuperSignal West Pico Chemiluminescent Substrate or SuperSignal West Femto Chemiluminescent Substrate (Thermo, Rockford, IL, USA) according our previously published methods^16^. The numbers in the figure indicated the Image J results of protein expression versus vector control.

### Statistical analysis

SPSS software 15.0 for windows (SPSS, Chicago, IL, USA) were used for data statistically analysis. The result values were showed as means ± standard deviations. The Kolmogorov–Smirnov test was used for the normality of the data. Pearson correlation matrix used for hierarchical gene analysis and heat maps for genetic association. According to the probability distribution pattern and the total number of cases and control, parametric or nonparametric were used for tests. Subgroup analyses were done by obesity level (BMI level) of subjects^37^. The significance level was set at *P* value less than 0.05.

## Supplementary information

Supplementary Table S1.

Supplementary Table S2.

Supplementary Figures and Table S3.

Supplementary Table S4.
