# Author Correction: *NT5C2* methylation regulatory interplay between DNMT1 and insulin receptor in type 2 diabetes

**DOI:** 10.1038/s41598-021-85727-z

**Published:** 2021-03-22

**Authors:** Yng‑Tay Chen, Wei‑De Lin, Wen‑Ling Liao, Ya‑Ching Tsai, Jiunn‑Wang Liao, Fuu‑Jen Tsai

**Affiliations:** 1 Graduate Institute of Food Safety, College of Agriculture and Natural Resources, National Chung Hsing University, Taichung, Taiwan; 2 Human Genetic Center, Department of Medical Research, China Medical University Hospital, China Medical University, Taichung, Taiwan; 3 School of Post Baccalaureate Chinese Medicine, China Medical University, Taichung, Taiwan; 4 Graduate Institute of Integrated Medicine, China Medical University, Taichung, Taiwan; 5 Center for Personalized Medicine, China Medical University Hospital, Taichung, Taiwan; 6 Graduate Institute of Veterinary Pathobiology, National Chung Hsing University, Taichung, Taiwan; 7 School of Chinese Medicine, China Medical University, Taichung, Taiwan; 8 Department of Health and Nutrition Biotechnology, Asia University, Taichung, Taiwan

Correction to: *Scientific Reports* 10.1038/s41598-020-71336-9, published online 30 September 2020

The original version of this Article contained errors.

In the Results, in subsection ‘DNA promoter methylation in patients with T2D’,

“Analysis of DNA methylation status was carried out using model-based analysis of tiling-arrays (MAT) levels, found 1,091 genes were hypermethylation in promoter regions.”

now reads:

“Analysis of DNA methylation status was carried out using model-based analysis of tiling-arrays (MAT) levels, found 1091 genes were hypermethylation in promoter regions (Table S4).”

The Article contained a reference to a circus plot, which was removed during revision of the manuscript prior to publication. In the corrected Results, in subsection ‘DNA promoter hypermethylation and gene expression in T2D mice’ the following sentence is now removed:

“We also performed a gene expression array result circus plot of KK-Ay mice with T2D and control mice using RNA samples and GeneChip Mouse Exon 1.0 ST arrays.”

Data supporting the statement that *FUT8* mRNA levels are not significantly different between t2D and healthy controls, was not included. This is now added as Supplementary Figure S2 in the accompanying Supplementary information files, and included in the Results section, in subsection ‘NT5C2 and FUT8 mRNA expression from peripheral blood of T2D patients’, where.

“However, *FUT8* mRNA levels in T2D were showed no significant difference with healthy controls.”

now reads:

“However, *FUT8* mRNA levels in T2D were showed no significant difference with healthy controls (Figure S2).”

The study uses a BMI of 24 as a normal weight/overweight threshold. The justification for this is now included in the Results, in subsection ‘Effects of NT5C2 on BMI’ where the following sentence was added in the first paragraph:

“In Taiwan, BMI categories included underweight (BMI < 18.5 kg/m^2^), normal weight (18.5 ≤  BMI < 24 kg/m^2^), overweight (24 ≤ BMI < 27 kg/m^2^), obesity (BMI ≥ 27 kg/m^2^).”

Data in the paper did not directly support the claim that NT5C2 is inhibited in mouse pancreatic β-cells specifically by DNMT1. Therefore, in the Results the title of section

‘NT5C2 was inhibited by DNMT1 in pancreatic β-cells in mice’

now reads:

‘NT5C2 was inhibited in pancreatic β-cells in mice’.

In the Results, in subsection ‘NT5C2 affected the insulin signaling pathway via DNMT1’ the following sentence, which provides previously missing information, is now added, where

“A plasmid containing a fragment of human DNMT1 or NT5C2 was constructed and transiently transfected into RIN-m5F cells.”

now reads:

“A plasmid containing human DNMT1 or NT5C2 was constructed and transiently transfected into RIN-m5F cells.”

The information on how the sampling for methylation analysis was done, was missing. It is now included in the methods section together with previously missing reference to the article describing MAT-score calculation method, which is now cited as Ref 34. Therefore, in Methods and materials, subsection ‘DNA methylation microarray analyses for human T2D and control’.

“The 40 samples (30 T2D cases and 10 healthy controls) which pass QC according principal component analysis. The methylation regions of the T2D and the controls were compared using the Model-based Analysis of Tiling-array (MAT) calculation (*P* < 0.001).”

now reads:

“The 40 samples (30 T2D cases were sample randomly from the 94 T2D patients and 10 healthy controls were sample randomly from the 98 healthy controls) which pass QC according principal component analysis. The methylation regions of the T2D and the controls were compared using the Model-based Analysis of Tiling-array (MAT) calculation (P < 0.001)^34^.”

Furthermore, there were multiple referencing errors in the Discussion section, where:

“NT5C2 is expressed in skeletal muscle from tissue expression study^25,26,27^. *NT5C2* in human myotubes, increases AMP-activated protein kinase (AMPK), acetyl-CoA carboxylase phosphorylation, and promotes lipid oxidation and glucose transport. The NT5C2 and AMPK activity in T2D and obesity may play an important role in the regulation of insulin action and lipid metabolism in skeletal muscle^28^.

NT5C2 is present in the brain, heart, skeletal muscle, erythrocytes, spleen, testis, fibroblasts, and endothelial cells^29^. We checked the NT5C2 protein expression of the brain, heart, muscle, spleen, and testis by immunohistochemistry staining. Since the NT5C2 were low activity presentations in these tissues, there was no significant difference between KK and KK-Ay mice (data not showed). KK-Ay mice with the lethal yellow obese (A^y^) mutation and develop diabetes of polygenic origin, showing severe obesity, hypertriglyceridemia, hyperglycemia, hyper-insulinemia, and insulin function loss by 42 weeks of age^18,21,30,31^. In our results, pancreatic β-cell mass was proliferation, and NT5C2 protein expression was inhibited in T2D mice’s pancreatic β-cell. Our previously results showed DNMT1 was significantly overexpression in T2D mice’s pancreatic β-cells^20,21^. Previously study have showed the importance of DNA methylation in pancreatic islet function^10^. In these studies, researchers identified the promoters of the insulin gene (*INS*)^32^; pancreatic and duodenal homeobox 1 (PDX1)^33^, which encodes a transcription factor important for both pancreatic development^34^ and the function of mature β-cells^35^; PPARG coactivator 1 alpha (*PPARGC1A*)^36^; and glucagon-like peptide 1 receptor (GLP1R)^37^, which stimulates insulin secretion and protects β-cell proliferation, as hypermethylated in islets from donors with T2D compared with those in islets from nondiabetic donors^38^. A hypermethylation was associated with reduced mRNA expression of the respective gene in pancreatic islets and with higher glycated HbA1c, suggesting the role in β-cell disturbs in T2D. High levels of glucose were also found to directly increase DNA methylation of *Pdx1* and *Ins* in clonal β-cells^32,33^.”

now reads:

“NT5C2 is expressed in skeletal muscle from tissue expression study^23^. *NT5C2* in human myotubes, increases AMP-activated protein kinase (AMPK), acetyl-CoA carboxylase phosphorylation, and promotes lipid oxidation and glucose transport. The NT5C2 and AMPK activity in T2D and obesity may play an important role in the regulation of insulin action and lipid metabolism in skeletal muscle^23^.

NT5C2 is present in the brain, heart, skeletal muscle, erythrocytes, spleen, testis, fibroblasts, and endothelial cells^23^. We checked the NT5C2 protein expression of the brain, heart, muscle, spleen, and testis by immunohistochemistry staining. Since the NT5C2 were low activity presentations in these tissues, there was no significant difference between KK and KK-Ay mice (data not showed). KK-Ay mice with the lethal yellow obese (A^y^) mutation and develop diabetes of polygenic origin, showing severe obesity, hypertriglyceridemia, hyperglycemia, hyperinsulinemia, and insulin function loss by 42 weeks of age^18,21,25,26^. In our results, pancreatic β-cell mass was proliferation, and NT5C2 protein expression was inhibited in T2D mice’s pancreatic β-cell. Our previously results showed DNMT1 was significantly overexpression in T2D mice’s pancreatic β-cells^20,21^. Previously study have showed the importance of DNA methylation in pancreatic islet function^10^. In these studies, researchers identified the promoters of the insulin gene (*INS*)^27^; pancreatic and duodenal homeobox 1 (PDX1)^28^, which encodes a transcription factor important for both pancreatic development^29^ and the function of mature β-cells^30^; PPARG coactivator 1 alpha (*PPARGC1A*)^31^; and glucagon-like peptide 1 receptor (GLP1R)^32^, which stimulates insulin secretion and protects β-cell proliferation, as hypermethylated in islets from donors with T2D compared with those in islets from nondiabetic donors^33^. A hypermethylation was associated with reduced mRNA expression of the respective gene in pancreatic islets and with higher glycated HbA1c, suggesting the role in β-cell disturbs in T2D. High levels of glucose were also found to directly increase DNA methylation of *Pdx1* and *Ins* in clonal β-cells^27,28^.”

Additionally, there were referencing errors in the Methods and materials section, in subsection ‘Subject participants’, where:

“T2D subjects were enrolled from China Medical University Hospital (CMUH)^39^ ”.

now reads:

“T2D subjects were enrolled from China Medical University Hospital (CMUH)^35^”.

In subsection ‘Animals’,

“The experiment according to the criteria for the care and use of KK and KK-Ay mice laid out in the ‘‘Guidebook for the Care and Use of Laboratory Animals’’^40^.”

now reads:

The experiment according to the criteria for the care and use of KK and KK-Ay mice laid out in the ‘‘Guidebook for the Care and Use of Laboratory Animals’’^36^.”

Lastly, in subsection ‘Statistical analysis’,

“Subgroup analyses were done by obesity level (BMI level) of subjects^41^.”

now reads:

“Subgroup analyses were done by obesity level (BMI level) of subjects^37^.”

The numerical values shown in the Venn diagram in Figure 1 were incorrect. The original version of Figure 1 appears below as Figure 1.

**Figure 1:**
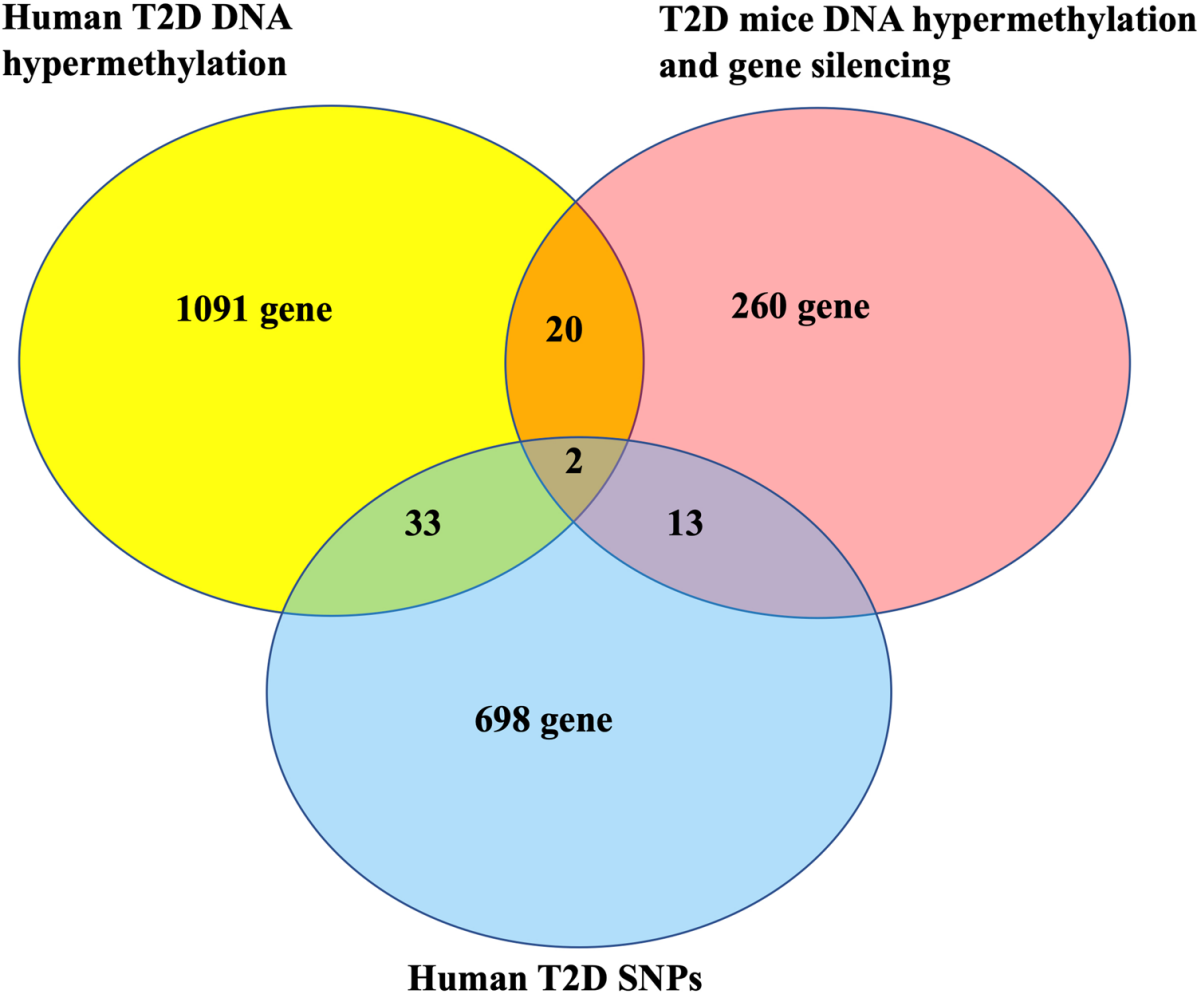
The original incorrect version of Figure 1.

The arrows in Figure 3 were not correctly aligned. The original version of Figure 3 appears below as Figure 2.

**Figure 2:**
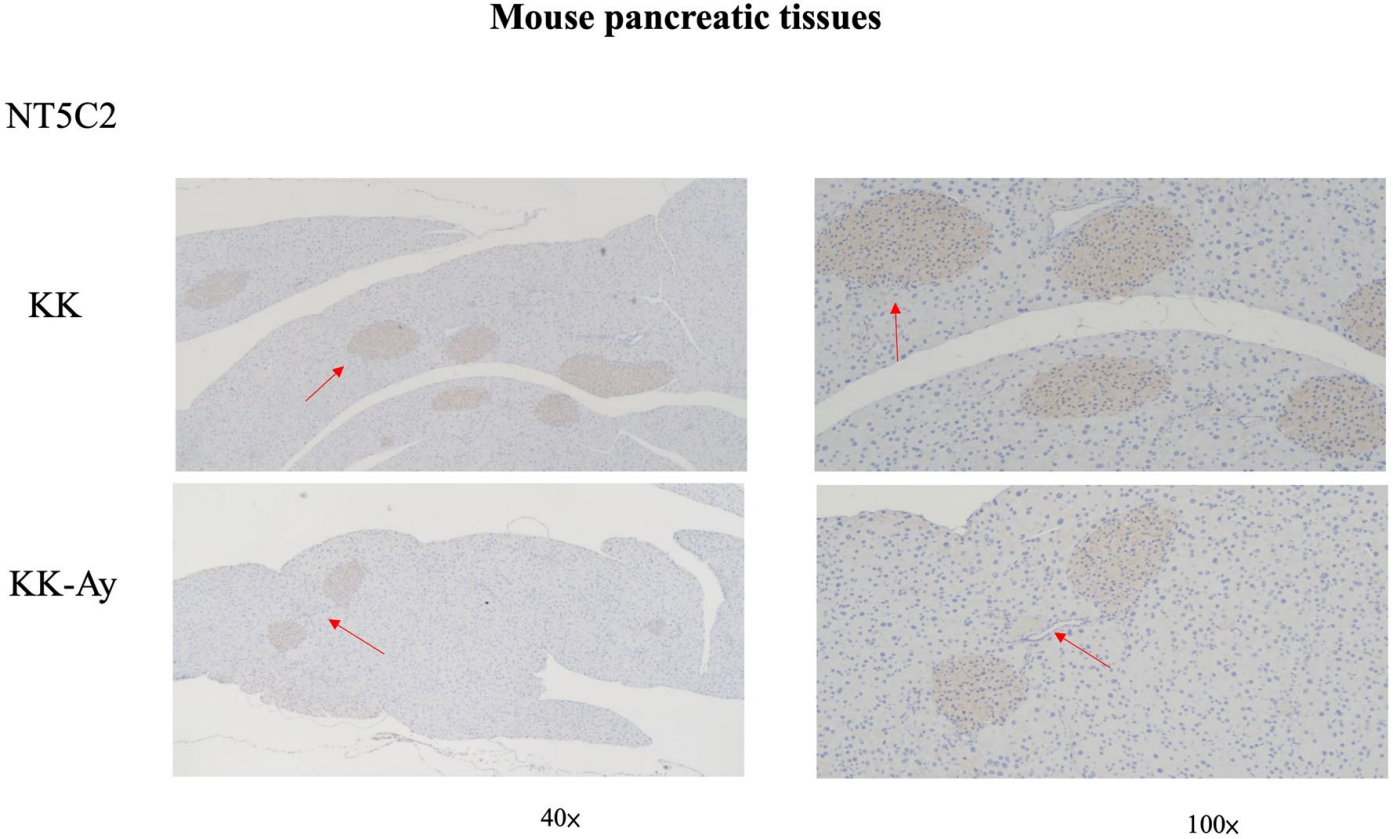
The original incorrect version of Figure 3.

In the legend of the same Figure 3, the following description is added for clarity: “The brown color depicts NT5C2-positive cells in the pancreas. Representative images of anti-NT5C2 IHC on the pancreas, the NT5C2 expression was higher in KK mice than KK-Ay mice. The red arrowheads are pointing at NT5C2-positive cells.”

The legend of Figure 5 was revised for accuracy, where

“DNMT1 epigenetically regulate NT5C2 and insulin receptor.”

now reads:

“DNMT1 epigenetically regulates NT5C2.”

The order of References 23–41 has changed and incorrectly cited references were removed, therefore

23. Careddu, M. G. *et al.* Knockdown of cytosolic 5′-nucleotidase II (cN-II) reveals that its activity is essential for survival in astrocytomacells. *Biochim. Biophys. Acta* **1783**, 1529–1535 (2008).
24. Hunsucker, S. A., Mitchell, B. S. & Spychala, J. The 5′-nucleotidases as regulators of nucleotide and drug metabolism. *Pharmacol. Ther.* **107**, 1–30 (2005).
25. Allegrini, S. *et al.* Bovine cytosolic IMP/GMP-specific 5′-nucleotidase: cloning and expression of active enzyme in *Escherichia coli*. *Biochem. J.* **328**, 483–487 (1997).
26. Rampazzp, C. *et al.* Human high-Km 5′-nucleotidase effects of overexpression of the cloned cDNA in cultured human cells. *Eur. J. Biochem.* **261**, 689–697 (1999).
27. Hanisch, F., Hellsten, Y. & Zierz, S. Ecto- and cytosolic 5′-nucleotidases in normal and AMP deaminase-deficient human skeletal muscle. *Biol. Chem.* **387**, 53–58 (2006).
28. Kulkarni, S. S. *et al.* Suppression of 5′-nucleotidase enzymes promotes AMP-activated protein kinase (AMPK) phosphorylation and metabolism in human and mouse skeletal muscle. *J. Biol. Chem.* **286**, 34567–34574 (2011).
29. Careddu, M. G. *et al.* Knockdown of cytosolic 5′-nucleotidase II (cN-II) reveals that its activity is essential for survival in astrocytoma cells. *Biochim. Bioohys. Acta* **1783**, 1529–1535 (2008).
30. Iwatsuka, H., Shino, A. & Suzuoki, Z. General survey of diabetic features of yellow KK mice. *Endocrinol. Jpn.* **17**, 23–35 (1970).
31. Castle, C. K., Colca, J. R. & Melchior, G. W. Lipoprotein profile characterization of the KKA(y) mouse, a rodent model of type II diabetes, before and after treatment with the insulin-sensitizing agent pioglitazone. *Arterioscler. Thromb. Vasc. Biol.* **13**, 302–309 (1993).
32. Yang, B. T. *et al.* Insulin promoter DNA methylation correlates negatively with insulin gene expression and positively with HbA(1c) levels in human pancreatic islets. *Diabetologia* **54**, 360–367 (2011).
33. Yang, B. T. *et al.* Increased DNA methylation and decreased expression of PDX-1 in pancreatic islets from patients with type 2 diabetes. *Mol. Endocrinol.* **26**, 1203–1212 (2012).
34. Jansson, J. *et al.* Insulin-promoter-factor 1 is required for pancreas development in mice. *Nature* **371**, 606–609 (1994).
35. Kaneto, H. *et al.* PDX-1 and MafA play a crucial role in pancreatic beta-cell differentiation and maintenance of mature beta-cell function. *Endocr. J.* **55**, 235–252 (2008).
36. Ling, C. *et al.* Epigenetic regulation of PPARGC1A in human type 2 diabetic islets and effect on insulin secretion. *Diabetologia* **51**, 615–622 (2008).
37. Hall, E. *et al.* DNA methylation of the glucagon-like peptide 1 receptor (GLP1R) in human pancreatic islets. *BMC Med. Genet.* **14**, 76 (2013).
38. Ma, X., Guan, X. & Hua, X. Glucagon-like peptide 1-potentiated insulin secretion and proliferation of pancreatic beta-cells. *J. Diabetes* **6**, 394–402 (2014).
39. Hsieh, A. R. *et al.* Lack of association of genetic variants for diabetic retinopathy in Taiwanese patients with diabetic nephropathy. *BMJ Open Diabetes Res. Care* **8**, e000727 (2020).
40. Yu, J. Y. L. *et al. A Guideline for the Care and Use of Laboratory Animals (in Chinese)* 3rd edn. (Chinese Society for the Laboratory Animal Science, Taiwan, 2005).
41. Mahajan, A. *et al*. Fine-mapping type 2 diabetes loci to single-variant resolution using high-density imputation and islet-specific epigenome maps. *Nat. Genet.* **50**, 1505–1513 (2018).

now read:

23. Kulkarni, S.S. *et al.* Suppression of 5′-nucleotidase enzymes promotes AMP-activated protein kinase (AMPK) phosphorylation and metabolism in human and mouse skeletal muscle. *J Biol Chem* **286**, 34567–34574 (2011).
24. Hunsucker, S.A., Mitchell, B.S. & Spychala, J. The 5′-nucleotidases as regulators of nucleotide and drug metabolism. *Pharmacol Ther* **107**, 1–30 (2005).
25. Iwatsuka, H., Shino, A. & Suzuoki, Z. General survey of diabetic features of yellow KK mice. *Endocrinol Jpn* **17**, 23–35 (1970).
26. Castle, C.K., Colca, J.R. & Melchior, G.W. Lipoprotein profile characterization of the KKA(y) mouse, a rodent model of type II diabetes, before and after treatment with the insulin- sensitizing agent pioglitazone. *Arterioscler Thromb Vasc Biol* **13**, 302–309 (1993).
27. Yang, B.T. *et al.* Insulin promoter DNA methylation correlates negatively with insulin gene expression and positively with HbA(1c) levels in human pancreatic islets. *Diabetologia* **54**, 360–367 (2011).
28. Yang, B.T. *et al.* Increased DNA methylation and decreased expression of PDX-1 in pancreatic islets from patients with type 2 diabetes. *Mol Endocrinol* **26**, 1203–1212 (2012).
29. Jansson, J. *et al.* Insulin-promoter-factor 1 is required for pancreas development in mice. *Nature* **371**, 606–609 (1994).
30. Kaneto, H. *et al.* PDX-1 and MafA play a crucial role in pancreatic beta-cell differentiation and maintenance of mature beta-cell function. *Endocr J* **55**, 235–252 (2008).
31. Ling, C. *et al.* Epigenetic regulation of PPARGC1A in human type 2 diabetic islets and effect on insulin secretion. *Diabetologia* **51**, 615–622 (2008).
32. Hall, E. *et al.* DNA methylation of the glucagon-like peptide 1 receptor (GLP1R) in human pancreatic islets. *BMC Med Genet* **14**, 76 (2013).
33. Ma, X., Guan, X. & Hua, X. Glucagon-like peptide 1-potentiated insulin secretion and proliferation of pancreatic beta-cells. *J Diabetes* **6**, 394–402 (2014).
34. Johnson WE, *et al*. Model-based analysis of tiling-arrays for ChIP-chip. *Proc Natl Acad Sci USA.* 15;**103**(33), 12457–62. (2006)
35. Hsieh, A.R. *et al*. Lack of association of genetic variants for diabetic retinopathy in Taiwanese patients with diabetic nephropathy. *BMJ Open Diabetes Res Care* **8**, e000727 (2020).
36. Yu, J.Y.L. et al. A guideline for the care and use of laboratory animals (in Chinese). 3rd ed. Taiwan, ROC: Chinese Society for the Laboratory Animal Science. 2005.
37. Mahajan, A. *et al*. Fine-mapping type 2 diabetes loci to single-variant resolution using high-density imputation and islet-specific epigenome maps. *Nat Genet* **50**, 1505–1513 (2018).

Finally, the Article did not include the list of 1091 differentially methylated genes identified in the study. These are listed in the new Supplementary Table 4, which now accompanies the Article.

All errors have now been corrected in the PDF and HTML versions of the Article, and in the Supplementary Information files that accompany the Article.

