# Supplementary material for: NT5C2 methylation regulatory interplay between DNMT1 and insulin receptor in type 2 diabetes
Source: Sci Rep. 2020 Sep 30;10:16087. doi: 10.1038/s41598-020-71336-9 (PMC7527562; doi:10.1038/s41598-020-71336-9)
Supplement: Supplementary file 3 — Supplementary Figures and Table S3. [file 41598_2020_71336_MOESM3_ESM.pdf]

***NT5C2* methylation regulatory interplay between DNMT1 and insulin receptor in type 2 diabetes**

Yng-Tay Chen[1,*], Wei-De Lin[2,3], Wen-Ling Liao[4,5], Ya-Ching Tsai[2], Jiunn-Wang Liao[6], Fuu-Jen Tsai[2,7,8,*]

[1]Graduate Institute of Food Safety, College of Agriculture and Natural Resources, National Chung Hsing University, Taichung, Taiwan.

[2]Human Genetic Center, Department of Medical Research, China Medical University Hospital, China Medical University, Taichung, Taiwan.

[3]School of Post Baccalaureate Chinese Medicine, China Medical University, Taichung, Taiwan.

[4]Graduate Institute of Integrated Medicine, China Medical University, Taichung, Taiwan.

[5]Center for Personalized Medicine, China Medical University Hospital, Taichung, Taiwan. Graduate

[6]Institute of Veterinary Pathobiology, National Chung Hsing University, Taichung, Taiwan.

[7]School of Chinese Medicine, China Medical University, Taichung, Taiwan.

[8]Department of Health and Nutrition Biotechnology, Asia University, Taichung, Taiwan.

[*]email: ytchen101@dragon.nchu.edu.tw; d0704@mail.cmuh.org.tw

Figure S1

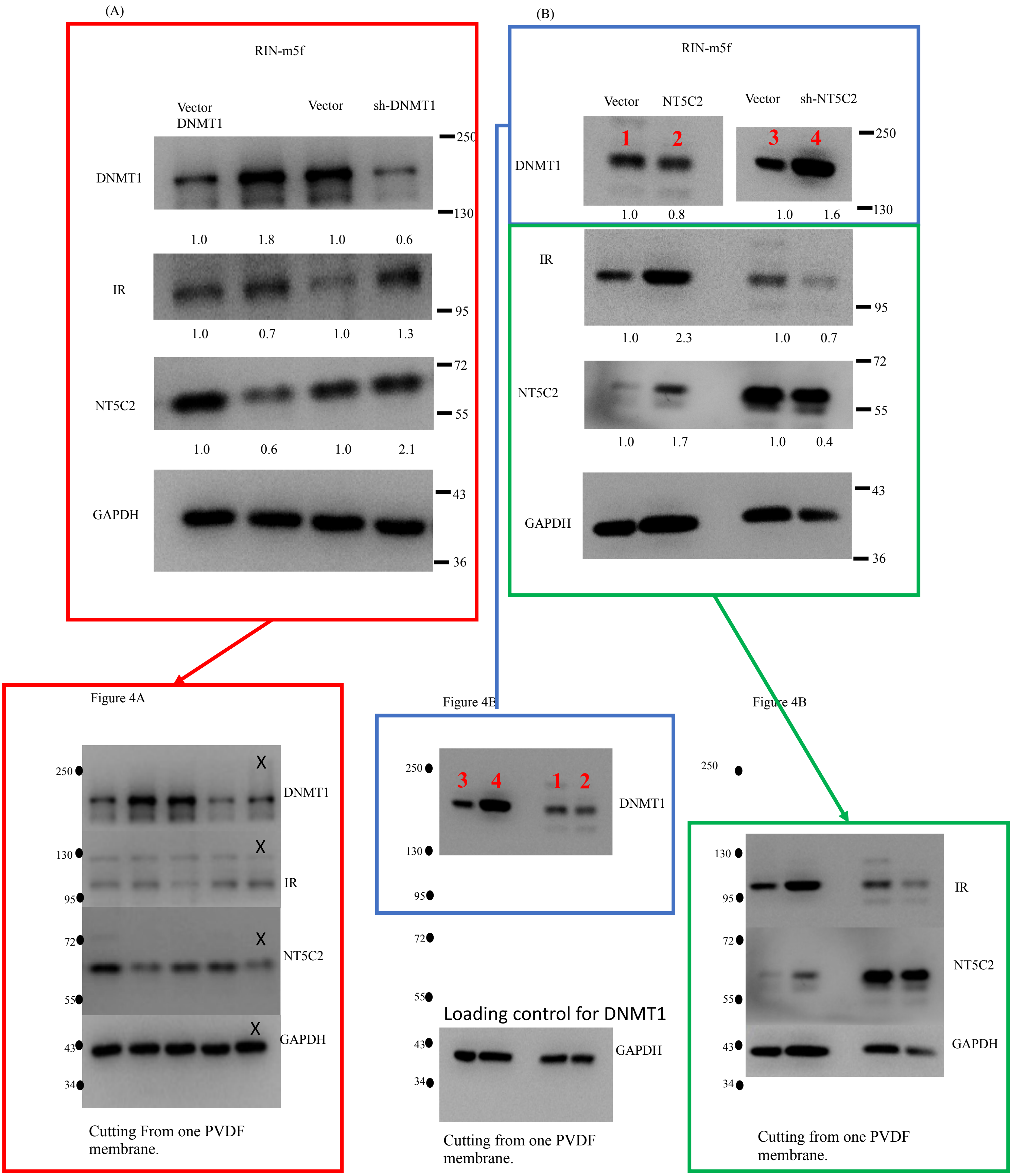
(A)
RIN-m5f
Vector
DNMT1
Vector
sh-DNMT1
DNMT1
250
130
1.0 1.8 1.0 0.6
IR
95
1.0 0.7 1.0 1.3
NT5C2
72
55
1.0 0.6 1.0 2.1
GAPDH
43
36
(B)
RIN-m5f
Vector
NT5C2
Vector
sh-NT5C2
DNMT1
1 2 3 4
250
130
1.0 0.8 1.0 1.6
IR
95
1.0 2.3 1.0 0.7
NT5C2
72
55
1.0 1.7 1.0 0.4
GAPDH
43
36
Figure 4A
250
DNMT1
130
IR
95
72
NT5C2
55
GAPDH
43
34
X
Cutting From one PVDF membrane.
Figure 4B
250
3 4 1 2
DNMT1
130
95
72
55
Loading control for DNMT1
GAPDH
43
34
Cutting from one PVDF membrane.
Figure 4B
250
130
IR
95
72
NT5C2
55
GAPDH
43
34
Cutting from one PVDF membrane.

Figure S2

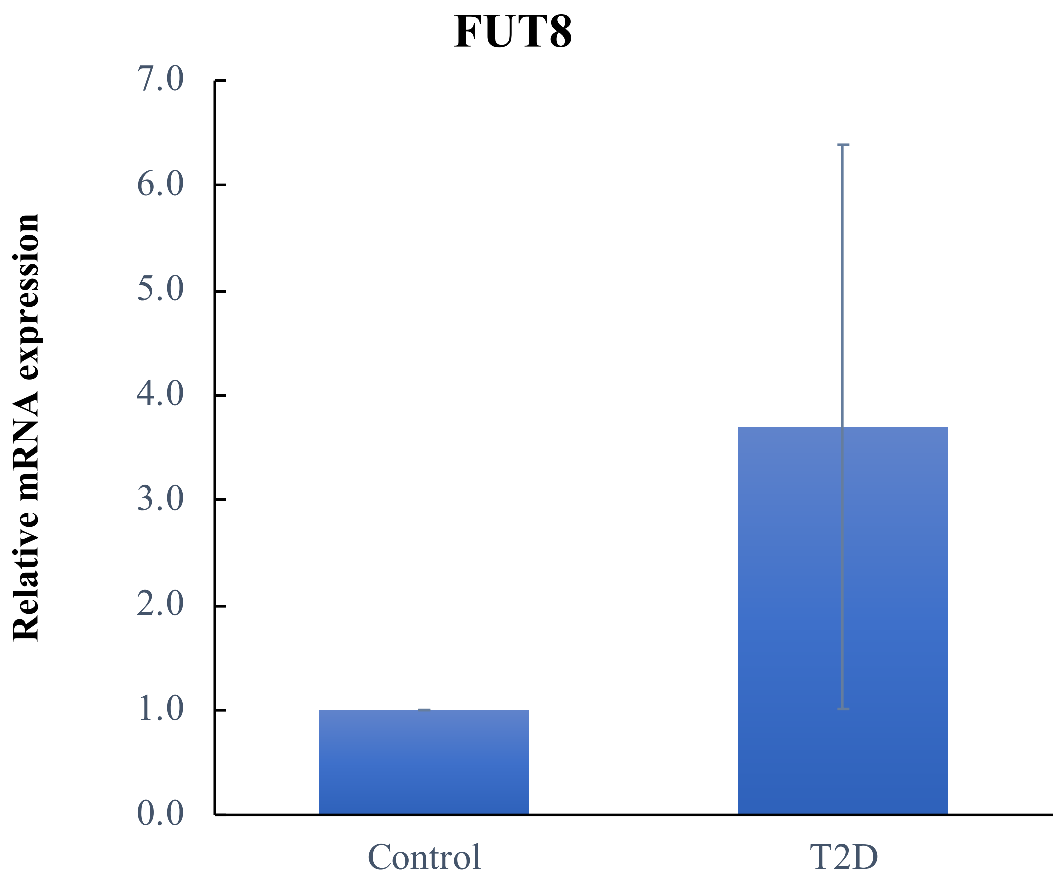

FUT8
Relative mRNA expression
7.0
6.0
5.0
4.0
3.0
2.0
1.0
0.0
Control
T2D

Supplementary Figure:

Figure S1. Original western blot panels with gel markers for Figure 4. The samples drive from the same experiment and the blots were processed in parallel. We were cutting the PVDF membrane according to molecular weight and reacted with DNMT1, NT5C2, IR, and GAPDH antibodies separately. The staining of GAPDH was used as a loading control.

Figure S2. FUT8 mRNA was non-significant difference between healthy control and patients with T2D. FUT8 mRNA expression is non-significant difference between patients with T2D (N=94) than in control (N=98). The relative mRNA levels in patients is 3.70 ± 2.69 versus healthy control ($P > 0.05$, SPSS software 15.0 for windows).

Supplementary Table:

Table S1. 698 genes with promoter and exon regions SNPs associated with T2D patients.

Table S2. 260 genes with promoter hypermethylated and gene silencing in KK-Ay mice. Gene expression array results were analyzed by using Affymetrix Expression Console software version 4.0. Genes and exons expression differences were identified by Transcriptome Analysis Console 2.0.0.9 software.

Table S3. The SNPs near NT5C2 gene which is associated with BMI in Asian and European population.

Table S4. The DNA promoter hypermethylated genes in T2D patients versus healthy control.

Table S3. The SNPs near NT5C2 gene which is associated with BMI in Asian and European population.

PUBMEDID	CHR_POS	STRONGEST SNP-RISK ALLELE	SNPS	RISK ALLELE FREQUENCY	P-VALUE	OR or BETA	95% CI (TEXT)	Population	MAPPED_TRAIT	STUDY ACCESSION
25673413	103109281	rs11191560-C	rs11191560	0.089	8.00E-09	0.031	[0.02-0.041] kg/m2 increase	European	body mass index	GCST002783
28443625	103109281	rs11191560-T	rs11191560	0.9015	7.00E-06	0.0371	[0.021-0.053] kg/m2 decrease	European	body mass index	GCST004499
28443625	103109281	rs11191560-T	rs11191560	0.9015	4.00E-06	0.0288	[0.017-0.041] kg/m2 decrease	European	body mass index	GCST004499
28443625	103109281	rs11191560-T	rs11191560	0.9015	5.00E-06	0.0337	[0.019-0.048] kg/m2 decrease	European	smoking behavior, body mass index	GCST004495
28443625	103109281	rs11191560-T	rs11191560	0.9015	5.00E-07	0.028	[0.017-0.039] kg/m2 decrease	European	smoking behavior, body mass index	GCST004495
28443625	103109281	rs11191560-T	rs11191560	0.9015	1.00E-07			European	smoking behavior, body mass index	GCST004497
28443625	103109281	rs11191560-T	rs11191560	0.9015	2.00E-06			European	smoking behavior, body mass index	GCST004497
24861553	103146454	rs11191580-C	rs11191580	0.27	4.00E-08	0.0295	[0.019-0.04] unit increase	East Asian	body mass index	GCST002461
25673413	103109281	rs11191560-C	rs11191560	0.091	2.00E-09	0.031	[0.021-0.041] kg/m2 increase	European	body mass index	GCST002783
26426971	103146454	rs11191580-T	rs11191580	0.9097	2.00E-11	0.031222157	unit decrease	European	body mass index	GCST005951
30108127	103109281	rs11191560-T	rs11191560	NR	8.00E-15	0.033	[NR] unit decrease	European	body mass index	GCST006368
